# COVID-19 Vaccination Protects Skeletal Muscle Against Statin-Related Side Effects

**DOI:** 10.3390/vaccines13040357

**Published:** 2025-03-27

**Authors:** Daria Schetz, Jacek Sein Anand, Karolina Kuźbicka, Marcin Wirtwein, Ivan Kocić

**Affiliations:** 1 Department of Pharmacology, Faculty of Medicine, Medical University of Gdańsk, 80-210 Gdańsk, Poland; 2 Department of Clinical Toxicology, Medical University of Gdańsk, 80-210 Gdańsk, Poland; 3 Pomeranian Centre of Toxicology, 80-104 Gdańsk, Poland

**Keywords:** COVID-19, statins, creatine kinase, muscle pain, vaccination, Pfizer–BioNTech, Comirnaty, myopathy, rhabdomyolysis

## Abstract

**Introduction**: COVID-19, caused by SARS-CoV-2, has disrupted global health systems, with vaccines being essential to mitigating its impact. Statins, widely prescribed for dyslipidemia, are associated with muscle-related side effects, which may worsen during COVID-19. This study explores the association between statin use, COVID-19 vaccination, and skeletal muscle-related symptoms. **Aims**: To evaluate the association between statin use and muscle symptoms (pain and creatine kinase (CK) levels) in COVID-19 patients and investigate whether vaccination is associated with changes in these symptoms. **Methods**: This observational study included 147 symptomatic COVID-19 patients: 74 chronic statin users (SG) and 73 non-users (CG). Vaccination status (unvaccinated, one-dose, or two-dose Pfizer–BioNTech) was recorded. Muscle pain was assessed using the Numerical Rating Scale (NRS), and CK levels were measured. Additional factors, including age, sex, BMI, and smoking status, were analyzed. Statistical tests examined the potential associations between statin use, vaccination, and muscle-related outcomes. **Results**: Higher CK levels were more frequently reported in SG, with severe rhabdomyolysis occurring slightly more often in the SG (4% vs. 3%). Men had higher CK values, while women appeared to be at greater risk of severe rhabdomyolysis. Older adults (≥65 years) in the SG had significantly higher CK levels. Fully vaccinated individuals had lower CK values and reported less muscle pain, while unvaccinated participants had the highest incidence of CK abnormalities and severe muscle pain. No significant differences in CK levels were observed between SARS-CoV-2 variants. **Conclusions**: Statin use was associated with elevated CK levels and increased muscle pain severity. Older adults and women appeared more susceptible to severe muscle complications. Full vaccination was linked to lower CK values and reduced muscle symptoms. Further research is needed to confirm these findings.

## 1. Introduction

COVID-19, caused by the SARS-CoV-2 virus, was first identified in December 2019 and rapidly became a global health crisis. The emergence of variants, including Delta and Omicron, markedly influenced the pandemic’s course, with Delta dominating cases in Poland through 2021 before being replaced by Omicron in early 2022 [1]. Vaccination played a pivotal role in controlling the pandemic, with the first vaccines, including Pfizer–BioNTech and Moderna, introduced in late 2020.

### 1.1. Mechanisms, Efficacy, and Safety of the Pfizer–BioNTech COVID-19 Vaccine

COVID-19 vaccines, including the Pfizer–BioNTech vaccine, are designed to stimulate both the humoral and cellular immune responses, primarily targeting the spike (S) protein of the SARS-CoV-2 virus. By producing neutralizing antibodies and activating T cells, the vaccine prevents viral entry into cells and mitigates systemic spread [2]. The Pfizer–BioNTech vaccine, developed and authorized at unprecedented speed, has been pivotal in reducing severe disease, hospitalization, and death, even against emerging variants like Delta and Omicron.

The mRNA technology underpinning the vaccine uses lipid nanoparticles to deliver the modified mRNA encoding the spike protein, ensuring efficient antigen expression and durable immunity. Studies confirm its ability to generate robust antibody and cellular responses, crucial for long-term protection [2]. While the efficacy against infection is lower for some variants, the vaccine consistently protects against severe outcomes.

### 1.2. Myopathies Associated with Statin Use: Symptoms, Mechanisms, and Management

Statins, or hydroxymethylglutaryl-coenzyme A (HMG-CoA) reductase inhibitors, are pivotal in managing dyslipidemia and reducing cardiovascular risk. Despite their proven benefits, statins are associated with muscle-related side effects, collectively termed statin-associated myopathy (SAMS).

SAMS encompasses a broad spectrum of symptoms. Myalgia, presenting as muscle pain and weakness without elevated creatine kinase (CK) levels, occurs in up to 13% of patients. Statin-induced toxic myopathy is typically mild, with CK levels below 1000 U/L, while autoimmune myopathy, a rarer condition, involves muscle necrosis and CK levels exceeding 1000 U/L [3]. Symptoms such as muscle tenderness and fatigue often worsen with exertion, and in rare cases, facial, neck, or throat muscle involvement may lead to ptosis, dysphagia, and speech difficulties, impacting quality of life and requiring medical attention [4].

Rhabdomyolysis, a severe form of statin-induced myopathy, involves muscle breakdown and the release of intracellular components like myoglobin, CK, and lactate dehydrogenase into the bloodstream. This life-threatening condition can cause acute kidney injury (AKI) in up to 25% of cases [5]. Key symptoms include muscle pain, weakness, and dark urine due to myoglobinuria, often accompanied by systemic complications.

CK, an intracellular enzyme found in skeletal muscle, myocardium, and the brain, is a sensitive marker of muscle injury. It is released into the bloodstream following muscle damage caused by drugs, hypoxia, or inflammation. Although no universal diagnostic threshold exists, CK levels 3 to 5 times the upper limit of normal (~1000 U/L) are commonly used as a diagnostic criterion [6]. However, CK elevation does not always correlate with the severity of muscle or renal injury. Complications include hyperkalemia, disseminated intravascular coagulation, and respiratory failure. Early recognition and aggressive management, including hydration, urine alkalization, and renal replacement therapy when necessary, are crucial for reducing morbidity and mortality.

Nontraumatic rhabdomyolysis is often linked to metabolic disturbances or external insults, with medications and infections playing important roles. Among medications, statins are mentioned, refs. [7,8], especially in patients with risk factors such as advanced age, polypharmacy, or drug interactions. In 2002, a clinical advisory was jointly issued by the American College of Cardiology (ACC); the National Heart, Lung, and Blood Institute; and the American Heart Association. This advisory defined statin-associated rhabdomyolysis as the presence of muscle symptoms accompanied by elevated CK levels, generally exceeding 11 times the upper limit of normal (that is, 2200 IU/L), indicative of myonecrosis. It also highlighted the association with increased serum creatinine levels consistent with pigment-induced nephropathy and myoglobinuria [9]. Clinically important myonecrosis is estimated to occur in approximately 0.5% of patients using statins [10]. Among nontraumatic rhabdomyolysis, infections, particularly viral myositis, are also important contributors. SARS-CoV-2 has been increasingly associated with rhabdomyolysis, highlighting the relevance of COVID-19 as a trigger for this condition [11].

Recent reports have indicated that COVID-19 vaccination, particularly with mRNA vaccines, has been associated with rare cases of rhabdomyolysis. The underlying mechanism remains unclear, but proposed pathways include mitochondrial dysfunction, systemic inflammatory responses, and endothelial impairment induced by the spike protein, which is present in both SARS-CoV-2 infection and vaccine-induced immune responses [12]. While the overall risk of vaccine-related rhabdomyolysis appears to be low, cases have been documented in individuals with predisposing factors, such as advanced age, polypharmacy, or conditions affecting cellular energy metabolism, including statin therapy.

Several factors predispose individuals to statin-induced myopathy, including advanced age (80+ years), female sex, low body mass, renal or hepatic dysfunction, hypothyroidism, and inflammatory muscle diseases. Concomitant use of certain medications, such as fibrates (particularly gemfibrozil), azoles, macrolides, verapamil, amiodarone, and grapefruit juice, can increase statin plasma concentrations, heightening the risk of myopathy. Excessive alcohol consumption and intensive physical activity also serve as contributing factors [13].

Statins may induce myotoxicity through various mechanisms, including disruptions in muscle cell organelles, inflammatory and immune reactions, and systemic effects such as electrolyte imbalances and reduced oxygen supply to the muscle tissue [14]. A key pathway involves isoprenoid depletion in the mevalonate pathway, as statins inhibit HMG-CoA reductase, reducing cholesterol synthesis and interfering with protein prenylation. This affects the proteins essential for muscle integrity, leading to symptoms like muscle pain, weakness, and, in severe cases, rhabdomyolysis [15].

Additionally, statins lower circulating Coenzyme Q10 (CoQ10) levels by 16–54%, which may impair mitochondrial function. While direct evidence of CoQ10 depletion in muscle remains inconclusive, supplementation has been suggested to alleviate statin-associated myopathy [16].

Another proposed mechanism involves altered cholesterol synthesis in muscle cell membranes, potentially affecting membrane stability, though hereditary conditions with impaired cholesterol synthesis do not typically result in myopathy, questioning this theory [15].

Despite these risks, statins remain widely used due to their proven cardiovascular benefits. Regular patient monitoring and individualized treatment strategies are essential to minimize the adverse effects while ensuring safe and effective therapy.

### 1.3. Muscle-Related Symptoms in COVID-19 Infection

COVID-19 is associated with musculoskeletal symptoms, including myalgia and muscle weakness, which vary in severity. Myalgia, characterized by muscle aches, occurs in a notable proportion of COVID-19 patients but is generally less prevalent than in influenza, where muscle pain is a key feature [17].

The exact mechanisms of muscle involvement in COVID-19 remain unclear, but SARS-CoV-2 may affect ACE2-expressing tissues, including skeletal muscles. Combined with systemic inflammation and immune activation, this can lead to muscle damage [18]. CK is a key biomarker of muscle injury used in COVID-19 and other conditions.

### 1.4. The Role of CK Measurement in Assessing Myopathy Risk in COVID-19 Patients

Monitoring CK levels is crucial not only for detecting muscle involvement but also for guiding treatment decisions, such as identifying cases where intensive care or additional interventions may be needed. Additionally, serial CK measurements can help track the resolution of muscle symptoms during recovery. The recovery of muscle function post-COVID-19 is variable, with many patients showing improvements within 2–3 months [19]. However, ongoing weakness in some individuals underscores the need for continued monitoring and rehabilitation.

The assessment of muscle involvement in COVID-19 patients, including CK level measurements and objective strength testing, is essential for a comprehensive understanding of the disease’s impact on the musculoskeletal system and for the effective management of affected individuals.

### 1.5. Research Gap and Study Rationale—Unexplored Interactions Between Statins, COVID-19 Vaccination, and Myopathy Risk

Despite the extensive research on the safety and efficacy of COVID-19 vaccines, as well as the well-documented muscle-related side effects of statins, the interaction between these two factors remains poorly understood. Statins are widely prescribed for managing dyslipidemia and reducing cardiovascular risk, yet their association with muscle symptoms such as myalgia and, in rare cases, rhabdomyolysis, has raised concerns, particularly during systemic infections like COVID-19. Moreover, while COVID-19 vaccination has been shown to mitigate severe disease outcomes, there is a lack of research exploring its potential impact on the incidence and severity of statin-associated muscle symptoms. This gap in the literature is particularly significant given the widespread use of statins and the global push for vaccination campaigns. Understanding how vaccination might influence muscle-related adverse effects in statin users could provide critical insights into optimizing therapeutic strategies and improving patient safety. The present study seeks to address this research gap by evaluating the interplay between chronic statin use, COVID-19 vaccination, and the prevalence and severity of muscle symptoms, including elevated CK levels, in symptomatic COVID-19 patients. By focusing on this underexplored area, this study aims to contribute valuable data to the broader understanding of pharmacological and immunological interactions in the context of the COVID-19 pandemic.

### 1.6. Key Findings from Previous Studies

SARS-CoV-2 mechanisms: The virus’s spike (S) protein, particularly its receptor-binding domain (RBD), is crucial for host cell infection, making it the primary target for vaccine-induced neutralizing antibodies and T-cell responses.

Statins and myopathy: Statins are highly effective in managing dyslipidemia; however, they can cause muscle-related side effects like myalgia and, rarely, rhabdomyolysis, particularly in high-dose regimens or with predisposing factors.

COVID-19 and muscle symptoms: COVID-19 can induce myalgia and muscle weakness, potentially exacerbated by the virus’s interaction with ACE2 receptors in skeletal muscles, as evidenced by elevated CK levels in severe cases.

Potential statin–COVID-19 interactions: Preliminary data suggest a possible link between chronic statin use and more severe muscle symptoms during COVID-19 infection, likely influenced by inflammation and metabolic disruptions.

Preliminary study insights: Previous research by the study team revealed significantly higher CK levels and muscle symptoms in statin users, especially in advanced age, laying the groundwork for investigating how vaccination modifies these effects [20].

### 1.7. Aim and Scope

#### 1.7.1. Aim

The aim of this study is to evaluate the relationships between statin use, muscle-related adverse effects, and COVID-19 vaccination status. Specifically, the study investigates the prevalence and severity of muscle symptoms such as elevated CK levels and muscle pain in chronic statin users compared with a control group. Additionally, it seeks to assess the potential protective role of COVID-19 vaccination in mitigating these adverse effects.

#### 1.7.2. Scope

This study focuses on the following key aspects:

Vaccination status: Examination of the distribution of COVID-19 vaccination (0, 1, or 2 doses) and its potential impact on muscle-related outcomes.

CK levels: Assessment of CK levels in statin users and non-users, categorized by vaccination status, to identify patterns of abnormal CK elevation and their clinical significance.

Muscle symptoms: Analysis of the prevalence and severity of muscle pain depending on statin use and vaccination status.

Potential confounding factors: Consideration of BMI and smoking status as potential contributors to CK elevation and muscle symptoms.

Statistical associations: Identification of significant relationships between vaccination status, muscle-related side effects, and statin use through robust statistical testing.

Clinical implications: Exploration of how vaccination may influence the management of statin-induced muscle symptoms, providing insights for optimizing treatment strategies.

The study’s findings aim to contribute to a better understanding of the interaction between statins and COVID-19 vaccination, offering potential guidance for clinicians in managing adverse muscle symptoms in statin users. Furthermore, the study sets the groundwork for future research on the protective effects of vaccines against drug-related toxicities.

## 2. Methods

### 2.1. Study Design and Population

From October 2021 until the end of January 2022, the Pomeranian Pharmacovigilance Centre (PPC) of the Department of Pharmacology at the Medical University of Gdańsk, in collaboration with the Department of Clinical Toxicology at the Medical University of Gdańsk, collected data on symptomatic patients diagnosed with COVID-19 (a positive result was obtained using the RT-PCR test). The study population consisted of 147 adult patients. They attended an initial medical visit, during which active COVID-19 infection was confirmed, and all necessary data for the study were collected. The study population was selected based on the presence of symptoms significant enough to warrant medical consultation, thereby reducing the likelihood of bias due to differences in care-seeking behavior between vaccinated and unvaccinated individuals. During the study period, access to healthcare was influenced by national health policies rather than individual discretion, ensuring that care-seeking tendencies did not unduly influence the study population. The severity of muscle pain and CK activity was evaluated in each patient.

Patients were divided into two groups:

The study group (SG)—Individuals on chronic statin monotherapy for at least 6 months who had not experienced adverse effects of statins in the past, such as muscle pain and increased CK levels (information obtained on the basis of a previously completed questionnaire).

The control group (CG)—Individuals not using statins, who did not use statins or other drugs in the past six months.

### 2.2. Variables

Variables that were taken into account in the study were the age of patients, sex, body mass index (BMI), smoking status, and COVID-19 virus variant.

To distinguish between COVID-19 cases caused by the Delta or Omicron variant, patients were classified based on the period in which they were infected. According to epidemiological data in Poland, the Delta variant was dominant from October 2021 through mid-December 2021, while Omicron emerged in December 2021 and rapidly became the prevalent variant in January 2022. Consequently, patients with confirmed infection from October 2021 to mid-December 2021 were classified as Delta cases. Patients infected from mid-December 2021 until the end of January 2022 were considered to have the Omicron variant.

#### 2.2.1. Vaccination Status Assessment

Only patients who were either unvaccinated or vaccinated exclusively with the Pfizer–BioNTech COVID-19 vaccine were included in the study to ensure consistency in evaluating the impact of vaccination on the outcomes. Before inclusion in the study, each patient was asked about vaccine tolerance. No study participant reported experiencing severe or burdensome adverse effects, including muscle-related symptoms, following vaccination. If such cases had been identified, these individuals would not have been eligible for our study, as their muscle symptoms would have been presumed to result from the vaccine rather than the interaction between statins and COVID-19. This approach ensured that our findings reflect the impact of vaccination and statin use on muscle symptoms rather than confounding factors related to vaccine intolerance. In both the SG and the CG, the vaccination status of each patient was recorded as one of the following:

Unvaccinated.

Vaccinated with one dose (Pfizer–BioNTech).

Vaccinated with two doses (Pfizer–BioNTech).

#### 2.2.2. Subjective Symptoms That Were Taken into Account in the Study

Muscle pain: In each patient, the severity of muscle pain was assessed based on a Numerical Rating Scale (NRS), a widely accepted tool recommended by the World Health Organization (WHO) for subjective pain evaluation. The scale ranges from 0 to 10, where 0 represents “no pain” and 10 indicates “the most severe pain imaginable”. This classification ensures a standardized approach to pain assessment, allowing for consistent documentation and comparison across different patients and clinical settings. Pain evaluation was based on the self-reported experience of participants, acknowledging the inherently subjective nature of pain perception.

In the study, the scores were interpreted as follows:

- Score 0—no pain; a complete absence of pain.
- Scores 1–6—moderate pain.
- Scores 7–10—severe pain (described as of extremely high intensity that limits the patient’s ability to move).

#### 2.2.3. Objective Findings

CK measurement:

The CK level was measured for each patient in both the study group SG and CG. Blood samples were collected under standardized conditions, following at least 8 h of fasting, to minimize variability in CK levels.

Procedure:

Blood collection: Venous blood samples were drawn from each patient using sterile techniques.

Sample handling: The samples were immediately processed and analyzed or stored at −80 °C if analysis could not be performed immediately.

CK analysis: Serum CK levels were measured at a licensed medical laboratory in Gdansk following standardized protocols and quality control procedures. The exact analytical equipment and method used were in accordance with the laboratory’s standard operating procedures, ensuring accuracy and reproducibility. Results were reported in units per liter (U/L).

Reference range and grouping: CK values were categorized into five groups to facilitate result interpretation:

Normal: 20–200 IU/L.

Slightly elevated: 200–600 IU/L.

Markedly elevated: >600–1000 IU/L.

Early-stage rhabdomyolysis: >1000–2200 IU/L.

Severe rhabdomyolysis: >2200 IU/L.

The threshold of CK > 2200 IU/L for rhabdomyolysis was selected based on the 2002 clinical advisory jointly issued by the American College of Cardiology (ACC); the National Heart, Lung, and Blood Institute; and the American Heart Association. This advisory defined statin-associated rhabdomyolysis as the presence of muscle symptoms accompanied by elevated CK levels exceeding 11 times the upper limit of normal (that is, >2200 IU/L), a level indicative of significant myonecrosis [9]. This definition provided a robust and clinically relevant framework for identifying cases of statin-induced rhabdomyolysis.

The categorization of CK values into discrete groups reflects the fact that, in the context of muscle injury, the absolute CK value is less important than the range in which it falls. Given the natural variability in CK levels and their interpretation in clinical practice, using predefined thresholds helps stratify the severity of muscle involvement and provides a more meaningful way to assess potential myopathy or rhabdomyolysis. This approach allows for a more standardized comparison between patient groups and facilitates clinical decision making based on established reference values.

Objective:

The CK measurement aimed to evaluate potential muscle injury or myopathy during the symptomatic phase of COVID-19 and to compare CK levels between the SG and CG groups. The categorization into four groups allowed for a more precise interpretation of the degree of muscle involvement.

### 2.3. Inclusion and Exclusion Criteria

Age > 18 years.

Chronic statin use for at least six months prior to the study.

No reported history of adverse muscle symptoms before COVID-19 diagnosis.

Only individuals who did not experience moderate or severe adverse effects following COVID-19 vaccination as well as muscle-related symptoms (e.g., muscle pain, muscle weakness) were included in the study. Mild adverse effects, such as pain at the injection site, were acceptable.

COVID-19 diagnosis confirmed by RT-PCR.

Patients taking statins only in the treatment of dyslipidemia (monotherapy).

### 2.4. Exclusion Criteria

Use of medications other than statins in the treatment of dyslipidemia.

Use of other drugs, with the exception of blood pressure medications that do not interact with statins.

Co-infection, e.g., with influenza or another virus.

Comorbid conditions: diabetes mellitus, poorly controlled hypertension, neuromuscular disorders, chronic kidney disease, autoimmune myopathies, uncontrolled hypothyroidism, liver diseases, or malignant neoplasms.

Diagnosed muscular diseases.

Individuals who experienced moderate or severe adverse effects following COVID-19 vaccination were excluded from the study.

Pregnancy or lactation.

Age under 18.

Patients with incomplete data, e.g., missing CK values.

### 2.5. Data Collection

Data were collected using standardized questionnaires and medical records. Variables included the following:

Patient demographics: age, sex, BMI, smoking status, COVID-19 variant.

COVID-19 vaccination status and number of doses (unvaccinated, one dose, two doses).

Muscle-related symptoms: subjective muscle pain (Numerical Rating Scale, NRS).

CK levels: measured during active infection (medical visit).

### 2.6. Statistical Analysis

Pearson’s chi-square tests, Kruskal–Wallis tests (followed by Mann–Whitney tests when significant), ordered logit regression, and descriptive statistics were applied to the data. A descriptive analysis was conducted to assess the demographic characteristics of the participants. Chi-square tests were used to examine the associations between statin use and the occurrence of myopathy (assessed through muscle pain, muscle weakness, and CK levels) in patients diagnosed with COVID-19. Kruskal–Wallis tests followed by Mann–Whitney tests were applied to explore the correlation between statin use and myopathy occurrence, the relationship between age and myopathy, and the association between vaccination status and myopathy. An ordered logit regression analysis was performed to investigate the relationship between CK levels and factors such as age, number of vaccine doses, and statin use.

A *p*-value of < 0.05 was considered statistically significant. All statistical analyses were conducted using STATISTICA Version 13.3 software with the Plus add-on package (StatSoft, Inc., 2017, Tulsa, OK, USA).

## 3. Results

### 3.1. Demographic and General Health Characteristics of Study Participants

#### 3.1.1. Demographic Characteristics

The study included 74 patients in the SG and 73 patients in the CG. The mean age of participants in the statin group was 68.2 ± 13.2 years, compared with 66.1 ± 11.7 years in the control group. A statistical analysis of the mean ages showed no significant difference between the two groups (t = 1.02, *p* = 0.309). The gender distribution consisted of 38 women and 36 men in the SG and 41 women and 32 men in the CG.

Initially, the study aimed to enroll an equal number of patients in both groups. However, after the study was completed, it was found that one patient in the control group had a comorbid condition that could have influenced the results by affecting CK levels, thereby confounding the analysis. Since this patient did not meet the inclusion criteria, they were excluded from the final analysis, resulting in a slight difference in group sizes. The data are presented in Table 1.

#### 3.1.2. Global Health Status Assessment

A subset of demographic variables related to global health status was assessed to explore the potential confounding factors influencing CK levels and muscle pain. Data on smoking status, body weight, and body mass index (BMI) were collected from 147 participants.

Smoking status: Among all study participants, 23% (34 individuals) were current smokers, while 46% (68 individuals) were former smokers who had quit smoking at least 12 months prior to study enrollment. The remaining 31% (45 individuals) had never smoked. The proportion of current smokers was slightly higher in the SG (25%) than in the CG (21%), while the proportion of former smokers was also slightly greater in the SG (48%) compared with the CG (44%); however, these differences were not statistically significant (*p* = 0.392).

Body weight and BMI: The proportion of participants classified as overweight (BMI = 25.0–29.9 kg/m^2^) was significantly higher in the SG group compared with the CG group (*p* = 0.047), while the prevalence of obesity (BMI ≥ 30.0 kg/m^2^) was similar in both groups.

In the SG group, 20% (15 individuals) had a normal BMI (18.5–24.9 kg/m^2^), while 53% (39 individuals) were classified as overweight, and 27% (20 individuals) had obesity.

In the CG group, a slightly higher proportion of participants had a normal BMI—27% (20 individuals)—while 40% (29 individuals) were overweight, and 33% (24 individuals) were classified as obese.

The statistically significant difference in overweight prevalence suggests that BMI may have been related to CK levels and skeletal muscle symptoms to some extent; however, the comparable rates of obesity between the groups indicate that excess body weight alone is unlikely to fully explain the observed differences.

### 3.2. Vaccination Status Among Statin Users and Non-Statin Users

#### 3.2.1. Number of Vaccinations in SG

Among participants in the SG, the distribution of vaccination status varied considerably. More than one-third of the participants (36%) were unvaccinated. A smaller proportion, 22%, had received only one vaccine dose, while the largest subgroup, 42%, was fully vaccinated with two doses. This distribution suggests that while the majority of statin users had received at least one dose of the vaccine, a substantial proportion remained unvaccinated.

#### 3.2.2. Number of Vaccinations in CG

In the control group, the proportion of unvaccinated individuals was slightly higher than in the statin group, with 42% of participants not having received any vaccine doses. Only a small fraction of individuals (5%) had received a single dose, while more than half of the participants (52%) were fully vaccinated with two doses. This indicates that a greater proportion of participants in the control group had completed the full vaccination regimen compared with the statin group.

### 3.3. Comparison of CK Levels Between SG and CG

#### 3.3.1. Creatine Kinase Levels in SG

In the statin group, normal CK levels were observed in 43% of participants. However, more than half of the individuals (56%) had elevated CK levels. Among them, 24% exhibited slightly elevated CK levels, while 28% had markedly elevated values. No cases of early-stage rhabdomyolysis were detected, but severe rhabdomyolysis was reported in a small subset of participants (4%). These findings suggest that CK elevations were common among statin users, with a notable proportion experiencing significant increases.

#### 3.3.2. Creatine Kinase Levels in CG

In the control group, normal CK levels were observed in the majority of participants (68%). Elevated CK levels were present in 32% of individuals, with 14% showing slightly increased values and 15% exhibiting markedly elevated CK levels. Similar to the statin group, no cases of early-stage rhabdomyolysis were detected, while severe rhabdomyolysis was identified in a small proportion of participants (3%). These results indicate that CK elevations were less frequent in the control group compared with the statin group.

### 3.4. Gender Differences in Creatine Kinase Levels

The analysis of CK levels revealed distinct differences between men and women. Among female participants, 45% had normal CK levels, while 32% exhibited slightly elevated values. Markedly elevated CK levels were observed in 16% of women, and severe rhabdomyolysis occurred in 8% of cases. In contrast, men showed a different distribution pattern, with fewer cases of slightly elevated CK levels (17%) but a substantially higher proportion (42%) experiencing markedly elevated CK levels. Notably, despite the higher prevalence of markedly elevated CK levels in men, severe rhabdomyolysis was observed exclusively in women.

A Kruskal–Wallis test revealed a significant difference in CK levels between men and women (*p* = 0.030). Post hoc Mann–Whitney U tests confirmed that men had significantly higher CK levels than women (*p* = 0.012), particularly in the categories of “slightly elevated” and “markedly elevated” CK levels. While the expected frequencies for each category were similar between genders, the observed distributions showed clear differences, especially in the “slightly elevated” and “markedly elevated” categories. These findings suggest that gender-related factors may influence the pattern of CK elevation, with men more likely to develop high CK levels but women being at a higher risk of severe rhabdomyolysis.

**Figure 1:**
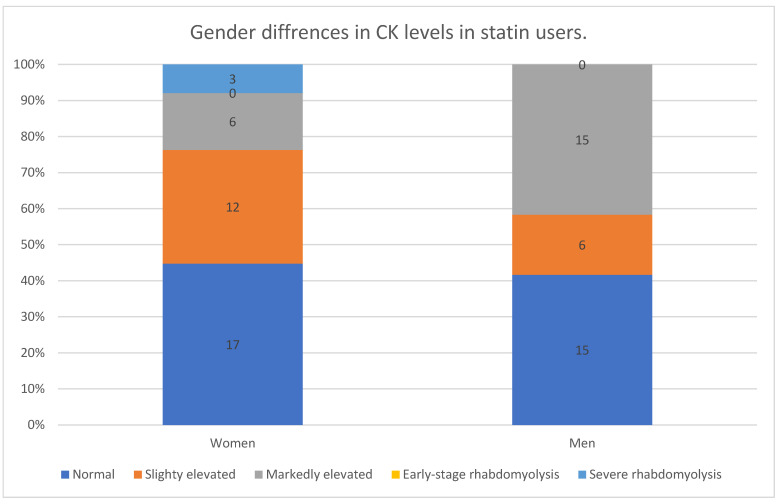
Gender differences in creatine kinase levels in statin users.

The data are presented in Figure 1.

#### 3.4.1. Association Between Statin Use and Elevated CK Levels

A Mann–Whitney U test showed that individuals taking statins (SG) had a significantly higher CK level (mean rank = 83.20) compared with those not using statins (mean rank = 64.68) (*p* = 0.003). For the purpose of reliable statistical analysis, patients with CK levels > 200–1000 IU/L (groups 2 and 3) were combined into a single category. This grouping was clinically justified, as statin-associated rhabdomyolysis is typically recognized at CK levels exceeding 1000 IU/L, and values below 2200 IU/L are less likely to indicate clinically significant myonecrosis.

#### 3.4.2. Age-Related Differences in CK Levels Among Statin Users

The analysis of CK levels in the study SG revealed differences across age categories. Among individuals aged 50 years or younger, most had normal CK levels, while a smaller proportion exhibited slightly or markedly elevated CK levels, and no cases of rhabdomyolysis were recorded. In the 51–65 age group, the distribution was similar, with a majority showing normal CK levels, followed by slightly and markedly elevated CK levels, and again, no cases of rhabdomyolysis. In contrast, participants aged 66 years or older had the highest prevalence of CK elevation, with a greater proportion displaying slightly and markedly elevated CK levels, and this was the only group in which rhabdomyolysis occurred.

Although the Kruskal–Wallis test did not reveal a statistically significant difference in CK distribution across all age groups (*p* = 0.551), the observed trend suggests that CK levels tended to be lower in younger individuals and higher in older individuals, particularly those aged 66 years or older. Further analysis using the Mann–Whitney U test, comparing individuals under and over 65 years old, showed that older individuals taking statins had significantly higher CK levels (mean rank = 80.21) compared with younger individuals (mean rank = 65.25) (*p* = 0.020).

These findings indicate that while age may not have a statistically significant impact on CK distribution when considering all age groups collectively, older statin users tend to have higher CK levels, with the oldest individuals being more prone to markedly elevated CK and statin-associated rhabdomyolysis. This trend suggests potential underlying biological or clinical factors that warrant further investigation.

**Figure 2:**
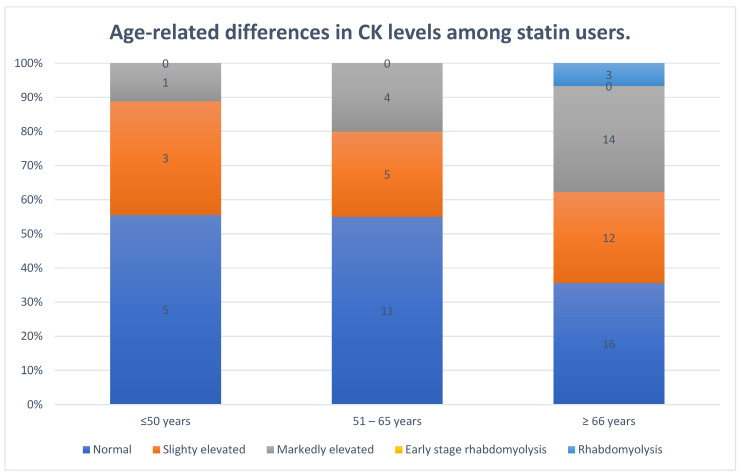
Age-related differences in creatinine kinase levels among statin users.

The data are presented in Figure 2.

### 3.5. CK Levels in SG and CG Based on Vaccination Status

A Kruskal–Wallis test and Mann–Whitney U tests revealed significant differences in CK levels between the vaccination groups (*p* < 0.001). The mean CK ranks were highest in the unvaccinated group (95.08) and lowest in the fully vaccinated group (51.50). Significant differences were found between unvaccinated patients and those fully vaccinated (*p* < 0.001). Individuals who received one dose had significantly higher CK levels than those who received two doses (*p* < 0.001) but did not differ significantly from unvaccinated individuals (*p* = 0.410).

#### 3.5.1. The Distribution of CK Levels in the SG, Categorized by Vaccination Status, Is Summarized as Follows

The distribution of CK levels among the statin users varied depending on vaccination status. In the unvaccinated group, only a small proportion (22%) had normal CK levels, while the majority exhibited some degree of CK elevation. Markedly elevated CK levels were particularly common, affecting nearly half of the participants (48%). Additionally, this was the only group in which rhabdomyolysis was reported, with three cases documented.

A similar pattern was observed among individuals who had received only one vaccine dose. The proportion of participants with normal CK levels remained low (25%), and a substantial number exhibited slightly (31%) or markedly elevated CK levels (44%). However, unlike the unvaccinated group, no cases of rhabdomyolysis were recorded.

In contrast, individuals who had received two vaccine doses showed a markedly different distribution. The majority (71%) had normal CK levels, and only a small fraction exhibited slightly (26%) or markedly elevated CK levels (3%). No cases of rhabdomyolysis were reported in this group.

**Figure 3:**
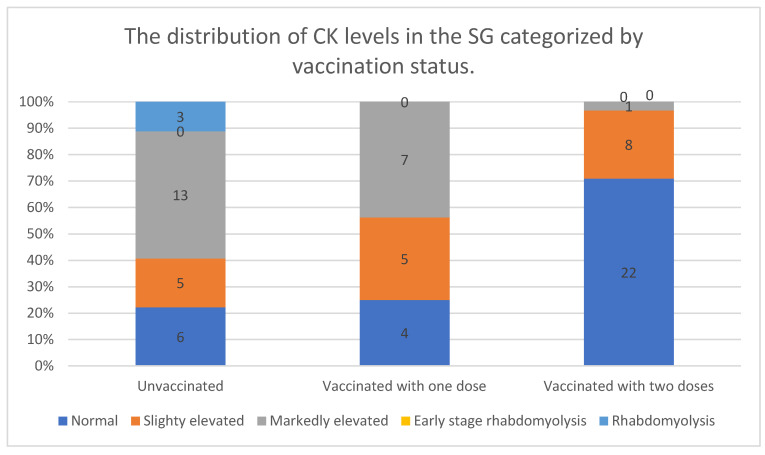
The distribution of CK levels in the SG, categorized by vaccination status.

These findings suggest a potential association between vaccination status and CK levels. Participants who were unvaccinated or had received only one dose were more likely to exhibit elevated CK levels, particularly in the markedly elevated range. Moreover, rhabdomyolysis was observed exclusively among unvaccinated individuals. In contrast, those who had received two vaccine doses had the lowest prevalence of CK elevation, with only one participant exhibiting markedly elevated CK levels. This trend may indicate a protective effect of full vaccination against significant CK elevations and statin-associated muscle damage. The data are presented in Figure 3.

#### 3.5.2. The Distribution of CK Levels in the CG, Categorized by Vaccination Status, Is Summarized as Follows

The distribution of CK levels in the CG varied depending on vaccination status. Among unvaccinated individuals, only 42% had normal CK levels, while the majority exhibited some degree of CK elevation. Markedly elevated CK levels were observed in 35% of unvaccinated participants, and two cases of rhabdomyolysis were reported exclusively in this group.

In contrast, individuals who had received one vaccine dose showed a different distribution pattern. The proportion of normal CK levels was significantly lower (33%), but no cases of markedly elevated CK or rhabdomyolysis were observed. Slightly elevated CK levels were recorded in two individuals.

The most notable trend was observed among fully vaccinated participants, where 90% had normal CK levels and only a small fraction (8%) exhibited slightly elevated CK levels. No cases of markedly elevated CK or rhabdomyolysis were recorded in this group.

These findings indicate that CK elevations and severe muscle damage were primarily observed in unvaccinated individuals, while those who had received at least one vaccine dose, particularly full vaccination, had a lower prevalence of CK abnormalities. The absence of markedly elevated CK levels and rhabdomyolysis in fully vaccinated individuals suggests a potential protective effect of vaccination against muscle damage associated with CK elevation.

**Figure 4:**
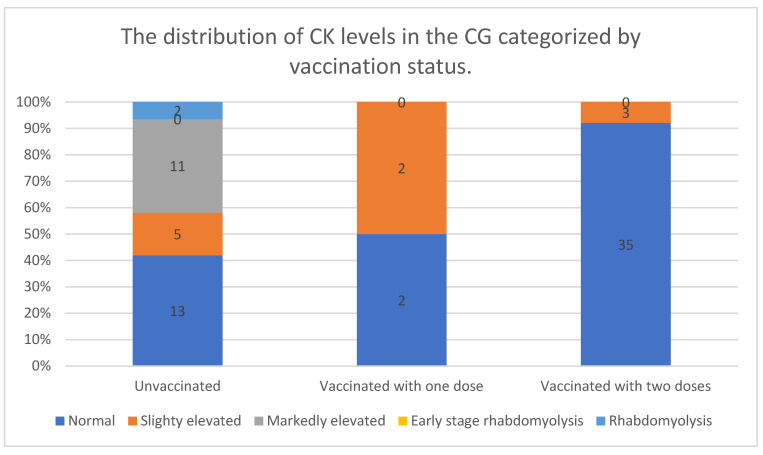
The distribution of CK levels in the CG, categorized by vaccination status.

The data are presented in Figure 4.

#### 3.5.3. Impact of Vaccination on CK Levels in the SG

A significant association was found between vaccination status (one or two doses) and CK levels in the SG group. CK levels were notably higher in unvaccinated patients compared with those who had received one or two vaccine doses.

To improve the reliability of the analysis, patients with CK levels between 200 and 1000 IU/L (groups 2 and 3) were merged into a single category. This classification was clinically justified, as early-stage rhabdomyolysis is typically diagnosed at CK levels above 1000 IU/L, while severe rhabdomyolysis is defined at levels exceeding 2200 IU/L.

Post hoc Mann–Whitney U tests were performed after the Kruskal–Wallis test to determine specific differences between groups. Among individuals taking statins, the Kruskal–Wallis test showed significant differences in CK levels between the vaccination groups (*p* < 0.001). The highest mean CK rank was observed in the unvaccinated group (48.83), while the lowest was in the fully vaccinated group (24.39).

The Mann–Whitney U tests confirmed that CK levels were significantly higher in individuals who received only one dose compared with those who were fully vaccinated (*p* < 0.001). Similarly, unvaccinated individuals had significantly higher CK levels than fully vaccinated participants (*p* < 0.001). However, there was no significant difference between unvaccinated individuals and those who received one dose (*p* = 0.295).

#### 3.5.4. Factors Influencing CK Levels: The Impact of Age, Statin Use, and Vaccination Status

The regression analysis revealed that certain variables were significantly associated with CK levels. Older individuals (≥65 years) who were unvaccinated and not using statins had a higher probability of elevated CK levels compared with younger individuals (<65 years) (*p* = 0.001, 95% CI [0.936, 3.774]). A more pronounced increase in CK levels was observed among older individuals who were unvaccinated but using statins (*p* < 0.001, 95% CI [1.559, 4.470]). Additionally, older individuals who had received one vaccine dose and were using statins also had significantly higher CK levels than the younger group (*p* = 0.006, 95% CI [0.682, 3.985]). Other interactions between variables, including the number of vaccine doses and statin use in the younger age group, did not reach statistical significance (*p* > 0.05), indicating that their effect on CK levels was not strong enough to be considered statistically significant in this model. A sensitivity analysis treating CK as a continuous variable in a multivariate regression model confirmed these findings, reinforcing that older age, statin use, and vaccination status were significant predictors of CK levels.

#### 3.5.5. Association of COVID-19 Variant with CK Levels in CG

To evaluate the impact of the COVID-19 variant (Delta vs. Omicron) on CK levels, an independent *t*-test was performed to compare CK values between patients infected with the Delta and Omicron variants. The study included 44 patients with the Delta variant (25 male, 19 female) and 29 patients with the Omicron variant (11 male, 18 female). The analysis revealed no statistically significant difference in CK levels between the two groups (*p* = 0.633). This indicates that the variant of the virus did not significantly affect muscle enzyme elevation in symptomatic COVID-19 patients.

#### 3.5.6. Association of COVID-19 Variant with CK Levels in SG

An independent *t*-test was conducted to examine whether the SARS-CoV-2 variant (Delta vs. Omicron) influenced CK levels in the statin group. Of these, 38 individuals (21 male, 17 female) were confirmed to have the Delta variant, while 36 (13 male, 23 female) were diagnosed with the Omicron variant. Statistical analysis did not reveal a significant difference in CK levels between these two subgroups (*p* = 0.518).

### 3.6. Comparison of Muscle Pain Between SG and CG

A comparison of muscle pain severity between the statin group and the control group revealed notable differences. Muscle pain was less frequently reported in the control group, where nearly half of the participants (46%) experienced no symptoms. In contrast, only 27% of individuals in the statin group reported an absence of muscle pain, suggesting a higher prevalence of discomfort in this group.

Moderate muscle pain was reported in similar proportions in both groups, affecting 32% of participants in the statin group and 37% in the control group. However, the most striking difference was observed in the occurrence of severe muscle pain. A significantly higher percentage of participants in the statin group (40%) reported experiencing severe muscle pain, whereas this was the case for only 16% of individuals in the control group.

These findings suggest that muscle pain, particularly severe symptoms, was more prevalent among statin users compared with non-users.

#### 3.6.1. Association Between Statin Use and Muscle Pain Severity

A statistically significant relationship was observed between statin use and the occurrence of muscle pain. Patients using statins reported moderate and severe muscle pain more frequently compared with non-users. The analysis revealed a significant difference, with a *p*-value of 0.003.

#### 3.6.2. Severity of Muscle Pain in SG Based on Vaccination Status

The severity of muscle pain among statin users varied depending on vaccination status, showing a trend toward reduced pain severity with a higher number of vaccine doses.

Muscle pain was most prevalent and severe among unvaccinated individuals, with the vast majority (74%) experiencing severe pain, while only a small proportion (15%) reported no muscle pain. Those who had received a single vaccine dose exhibited a slightly different pattern; severe muscle pain was still common (50%), but moderate pain was more frequent (38%) compared with unvaccinated individuals.

A notable shift was observed in fully vaccinated participants, where nearly half (45%) reported no muscle pain, and only a small percentage (6.5%) experienced severe pain. Moderate pain was the most common symptom in this group, affecting 48% of participants.

**Figure 5:**
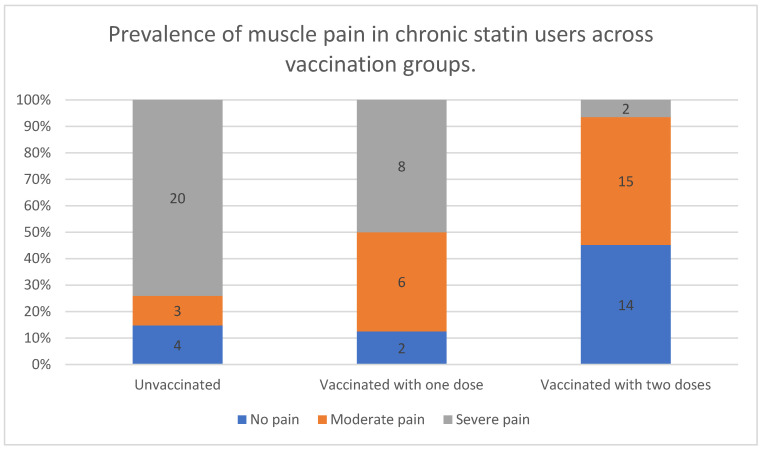
Prevalence of muscle pain in chronic statin users across vaccination groups.

These findings suggest a potential association between vaccination status and muscle pain severity, with fully vaccinated individuals experiencing significantly lower rates of severe pain compared with those who were unvaccinated or had received only one dose. However, the intermediate group, with a single vaccine dose, displayed a more variable pattern, with a higher frequency of moderate pain compared with the other groups. The data are presented in Figure 5.

#### 3.6.3. Impact of Vaccination Status and Dose on Muscle Pain in Statin Users

Relationship between vaccination status and muscle pain in SG:

A significant relationship was observed between vaccination status (1–2 doses) and the occurrence of muscle pain among statin users. Patients who received one or two doses of the vaccine reported moderate and severe muscle pain less frequently compared with unvaccinated individuals. The analysis revealed a statistically significant association with a *p*-value of 0.0001. However, it is important to note that some subgroups contained fewer than five participants, which limits the reliability of the statistical test.

Relationship between vaccination doses and muscle pain in SG:

An observed trend suggests a relationship between the number of vaccine doses received and the prevalence of muscle pain in statin users. Patients who received more vaccine doses reported muscle pain (moderate and severe) less frequently. Statistical analysis revealed a significant association with a *p*-value of 0.0001. However, due to the limited sample size in some subgroups (fewer than five participants), the reliability of this test is limited, and these results should be interpreted with caution.

## 4. Discussion

Understanding the balance between therapeutic benefits and potential risks is crucial in pharmacotherapy, particularly during global health crises such as the COVID-19 pandemic. Statins, widely used for cardiovascular protection, exemplify this complexity, as their benefits may sometimes be accompanied by adverse effects. One of the most notable concerns is statin-induced myopathy, which ranges from mild muscle pain and weakness to severe conditions like rhabdomyolysis. These adverse effects have been linked to genetic predisposition, drug interactions, and metabolic disruptions. Given the systemic impact of COVID-19 and its potential to exacerbate muscle-related complications, questions have arisen about the interplay between SARS-CoV-2 infection, statin therapy, and muscle health.

The role of statins in COVID-19 remains a topic of debate. Some studies highlight their potential benefits in mitigating disease severity [21,22], while others underscore the risks, such as statin-induced necrotizing autoimmune myopathy (SINAM), which can manifest even after discontinuation of therapy [23]. It remains unclear whether SARS-CoV-2 exacerbates statin-related muscle symptoms or if statins amplify the virus’s detrimental effects on skeletal muscles. This uncertainty led us to investigate whether statins increase the risk of myopathy in COVID-19 patients, especially given that muscle weakness correlates with respiratory compromise—a critical concern in severe cases of the disease.

Our previous study involved 66 COVID-19 patients divided into 2 groups: statin users without prior adverse effects and a control group of individuals who had not used any medications in the past 6 months. Results demonstrated that muscle pain and weakness—assessed via dynamometer-measured grip strength—were more frequent and severe in statin users. Additionally, higher CK levels were observed in this group, often correlating with the intensity of muscle pain [20]. These findings suggest a possible association between COVID-19 and an increased prevalence of statin-related muscle symptoms, particularly in older patients. However, without randomized trials comparing statin continuation versus discontinuation in COVID-19 patients, no definitive conclusions can be drawn regarding the optimal clinical approach.

While our previous study did not assess the impact of COVID-19 vaccination on myopathy risk in statin users, this remains a critical question. Both statins and COVID-19 infection can independently contribute to muscle-related complications through shared mechanisms, including mitochondrial dysfunction, oxidative stress, and immune-mediated muscle injury. Necrotizing autoimmune myositis (NAM) has been reported following both statin use and COVID-19 infection, suggesting overlapping pathophysiology. A recent review [24] discusses these similarities, emphasizing the need for further research into vaccine-induced immune activation and its potential role in muscle autoimmune phenomena. Case reports and pharmacovigilance data have documented instances of rhabdomyolysis following COVID-19 vaccination, as well as after COVID-19 infection itself. A review of VAERS data identified 386 cases of vaccine-associated rhabdomyolysis, though the self-reported nature of these cases limits causal inference. Additionally, multiple reports and systematic reviews have described viral myopathy and COVID-19-related rhabdomyolysis as potential complications of SARS-CoV-2 infection, highlighting the need for further investigation into the interplay between infection, vaccination, and muscle injury [25].

Our findings indicate an association between statin use and higher CK levels as well as increased severity of muscle pain in COVID-19 patients. Notably, fully vaccinated statin users exhibited lower CK levels and reduced muscle pain severity compared with their unvaccinated counterparts. One possible explanation is that vaccination mitigates systemic inflammation, a key driver of both statin-related myopathy and COVID-19-associated muscle symptoms. However, given the observational nature of our study, causality cannot be established, and further research is needed to clarify these relationships.

Several factors may influence CK levels and muscle symptoms, including BMI and smoking status [26]. While smoking prevalence did not differ significantly between groups, overweight status was more frequent in the statin group, reaching statistical significance. This suggests that BMI may have contributed to CK elevations and muscle symptoms, though the similar prevalence of obesity in both groups indicates that excess body weight alone cannot fully explain the observed differences.

Furthermore, we observed that women exhibited a higher proportion of slightly elevated CK levels compared with men (32% vs. 17%), whereas men demonstrated a higher prevalence of significantly elevated CK levels (42% vs. 16%). Additionally, all cases of statin-associated rhabdomyolysis in our study occurred in women (8%), suggesting potential sex-specific susceptibilities to severe muscle-related adverse effects. These differences may be attributed to variations in muscle mass, enzyme activity, and statin pharmacokinetics [27].

Age also played a role, as the oldest group (≥ 66 years) exhibited the highest CK variability and all cases of statin-associated rhabdomyolysis. Age-related changes in muscle metabolism, decreased medication clearance, and heightened inflammatory responses may contribute to this increased susceptibility.

Interestingly, our study found that CK levels did not significantly differ between symptomatic patients infected with the Delta and Omicron variants. Given that Omicron is generally associated with lower systemic inflammation than Delta [27], a difference in CK levels might have been expected. However, our findings suggest that muscle enzyme elevations in COVID-19 patients are more likely driven by factors such as systemic inflammation, pre-existing conditions, or medication use rather than specific viral variants. This underscores the need for further research into the interplay between COVID-19, statin therapy, and muscle injury.

Given the widespread use of statins and the ongoing impact of COVID-19, our findings highlight the need for further research into the potential interactions between statins, COVID-19, and vaccination. Future prospective studies could help determine whether vaccination plays a modifying role in statin-associated muscle symptoms and explore alternative explanations for the observed trends.

An alternative explanation for our findings could be that individuals who experienced notable muscle symptoms after their first vaccine dose chose not to receive the second dose, thereby creating a selection bias that artificially enhanced the apparent protective effect of two-dose vaccination. However, our data do not support this scenario. First, we included only participants who reported no clinically significant adverse muscle events following vaccination, making it unlikely that severe myalgias after the first dose systematically excluded individuals from our study. Moreover, we observed no substantial differences in baseline characteristics—such as age, sex, or body mass index—between the one-dose and two-dose groups that would suggest only “healthier” or less susceptible individuals proceeded to their second injection.

Additionally, the national vaccination strategy during our study period generally encouraged eligible individuals to complete the full vaccination regimen, and discontinuation was often influenced by factors unrelated to musculoskeletal side effects. If a large number of patients had developed severe muscle complaints post–first dose, this would likely have been reflected in local adverse event reports. As our data showed no such pattern, it appears more plausible that the lower CK levels and reduced frequency of severe muscle pain seen among the two-dose group reflect a genuine protective benefit of full vaccination rather than a simple artifact of patient self-selection.

Our observational findings suggest that COVID-19 vaccination could be associated not only with a milder course of infection, but also with fewer myopathy-related events in statin users. If confirmed by prospective or randomized studies, this association might help address vaccine hesitancy by indicating possible additional benefits beyond infection prevention. A potential explanation is that vaccination may lessen systemic inflammation—a key factor implicated in both statin-induced myopathy and COVID-19-related muscle injury. Moreover, the stepwise relationship between the number of vaccine doses and reduced muscle symptoms observed in this study could indicate that achieving full vaccination status might be important in minimizing inflammation-driven complications. However, experimental or longitudinal designs are needed to clarify whether this relationship is causal.

Future research should investigate whether different vaccine platforms (e.g., vector-based vaccines like AstraZeneca or Johnson & Johnson) yield comparable associations. Additionally, evaluating the impact of booster doses, heterologous vaccination regimens, and relevant population-specific factors (e.g., age, sex, comorbidities) could offer deeper insight. Ultimately, optimizing vaccination approaches in statin users may help maximize protection from severe COVID-19 manifestations while potentially mitigating muscle-related adverse events.

## 5. Study Limitations

This study has several limitations that should be considered when interpreting the findings. The relatively small sample size limits the generalizability of the results to the broader population. The inclusion criteria were highly restrictive, focusing on individuals without certain comorbidities (as listed in the exclusion criteria), vaccinated exclusively with the Pfizer–BioNTech vaccine (BNT162b2), on chronic statin monotherapy, and actively symptomatic with COVID-19. While these strict criteria ensured a controlled study population, they may not fully represent the diverse range of statin users in real-world settings, particularly those with comorbidities or polypharmacy.

Additionally, the study population consisted of individuals who sought medical attention for symptomatic COVID-19, excluding asymptomatic cases. This specificity limits the applicability of the findings, as asymptomatic individuals may experience different outcomes. Furthermore, while no significant demographic or clinical differences were observed between vaccinated and unvaccinated patients within the statin group, the inclusion of only symptomatic patients introduces the possibility of selection bias related to care-seeking behavior.

Vaccination status was limited to individuals receiving the Pfizer–BioNTech vaccine, restricting the ability to assess the potential effects of other vaccines on muscle-related symptoms in statin users. The observational nature of the study precludes definitive conclusions about causation. Although statistically significant associations were observed, these do not establish direct causal links between statin use, vaccination, and muscle-related symptoms during COVID-19 infection.

Another limitation is that CK levels were available only in categorical ranges. While this approach aligns with clinical practice, using continuous CK measurements could yield more precise statistical correlations. Future studies incorporating exact CK values could provide further insights.

No correction for multiple comparisons was applied, so all *p*-values below 0.05 should be interpreted with caution. Additionally, while CK levels and muscle symptoms were measured both objectively and subjectively, other potential biomarkers of muscle damage or systemic inflammation were not assessed, which might have provided additional insights into the mechanisms underlying the observed associations.

Despite these limitations, our findings provide valuable insights into the interaction between statin use, COVID-19 infection, and vaccination. Further research with larger and more diverse populations is needed to validate and expand upon these results.

## 6. Conclusions

The study findings indicate that both the SG and CG were broadly similar in terms of mean age and gender distribution. However, in the SG, a significantly higher proportion of participants was classified as overweight (BMI = 25.0–29.9 kg/m^2^) than in the CG. This difference in BMI profile suggests that excess body weight may be associated with differences in CK levels and muscle-related symptoms, although the comparable obesity rates across both groups imply that weight alone cannot fully explain the observed outcomes. Smoking status did not vary significantly between the SG and CG, indicating that tobacco use was unlikely to be a major confounding factor.

An essential observation was that elevated CK levels were more frequently reported in the SG. Both moderate and marked elevations were common among statin users, and although severe rhabdomyolysis (CK > 2200 IU/L) was uncommon in both groups, it was slightly more frequent in the SG (4% compared with 3% in the CG). Statistical analysis using a Mann–Whitney U test supported these findings by indicating higher CK levels in statin users than in non-users.

Further evaluation of gender-specific results suggested that men more frequently had markedly elevated CK values, whereas women had fewer markedly elevated levels but appeared to be at higher risk of severe rhabdomyolysis. This pattern emerged from Kruskal–Wallis tests and subsequent post hoc analyses, which indicated that, overall, men had higher CK levels, but the most severe muscle complications were more frequently observed in women.

Although an initial Kruskal–Wallis test across multiple age ranges within the SG did not yield statistical significance, dividing participants into two cohorts (<65 years vs. ≥65 years) showed that older patients had significantly higher CK values. Indeed, cases of severe rhabdomyolysis in the SG were confined to the oldest subgroup (≥66 years), suggesting that advancing age may be associated with greater susceptibility to muscle damage in statin users.

The study also examined vaccination status in relation to CK levels and muscle pain. Among both statin users and controls, fully vaccinated individuals (two doses) had the lowest CK levels and were less likely to experience severe muscle damage, including rhabdomyolysis. In contrast, unvaccinated participants had the highest incidence of elevated CK values and the greatest frequency of severe muscle complications, while those receiving only one dose showed intermediate results. This pattern was also observed in analyses of muscle pain severity. While unvaccinated statin users most frequently reported severe pain, the fully vaccinated more commonly described no pain or moderate discomfort. Consequently, the data suggest that full vaccination may be associated with reduced muscle damage, especially in statin users, although further research is needed to confirm this relationship.

When examining muscle pain across the two main groups, participants in the CG were more likely to report no pain symptoms. In that group, 46% reported no muscle pain, compared with only 27% of those in the SG. The most striking difference was in severe pain; 40% of statin users reported experiencing significant muscle discomfort compared with 16% of the control group. These findings suggest an association between statin therapy and increased muscle pain frequency and severity.

Regarding the potential influence of different variants of SARS-CoV-2 on CK levels, no statistically significant differences were observed between the Delta and Omicron variants in the CG and SG, suggesting that variant type was not associated with substantial differences in enzyme elevation in symptomatic COVID-19 cases.

Finally, regression analyses indicated that older adults (≥65 years) who were unvaccinated and taking statins were associated with a greater likelihood of having markedly elevated CK values. Conversely, full vaccination (two doses) was associated with lower muscle pain severity and a reduced incidence of CK abnormalities across multiple comparisons.

The authors confirm that the principal investigator for this paper is Daria Schetz and that she had direct clinical responsibility for the patients.

## Figures and Tables

**Table 1:** Demographic, clinical, and laboratory characteristics of chronic statin users and control group in relation to COVID-19 vaccination and muscle symptoms.

Variables	Chronic Statin Users N = 74		Control N = 73	
**Age (Mean ± S.D.)**	**68.2 ± 13.2**		**66.1 ± 11.7**	
**Women/Men**	**38/36**		**41/32**	
**CK levels**	**N**	**%**	**N**	**%**
Normal (20–200 IU/L).	32	43	50	68
Slightly elevated (200–600 IU/L)	18	24	10	14
Markedly elevated (>600–1000 IU/L)	21	28	11	15
Early-stage rhabdomyolysis (>1000–2200 IU)	0	0	0	0
Severe rhabdomyolysis (>2200 IU/L)	3	4	2	3
**Muscle pain**	**N**	**%**	**N**	**%**
No pain	20	27	34	46
Moderate	24	32	27	37
Severe	30	40	12	16
**Vaccination dose**	**N**	**%**	**N**	**%**
Unvaccinated	27	36	31	42
Vaccinated with 1 dose	16	22	4	5
Vaccinated with 2 doses	31	42	38	52

## Data Availability

Data supporting the findings of this study are available from the corresponding author on a reasonable request.
